# Diagnosis, treatment, and management of rickets: a position statement from the Bone and Mineral Metabolism Group of the Italian Society of Pediatric Endocrinology and Diabetology

**DOI:** 10.3389/fendo.2024.1383681

**Published:** 2024-04-19

**Authors:** Giampiero I. Baroncelli, Pasquale Comberiati, Tommaso Aversa, Federico Baronio, Alessandra Cassio, Mariangela Chiarito, Mirna Cosci o di Coscio, Luisa De Sanctis, Natascia Di Iorgi, Maria Felicia Faienza, Danilo Fintini, Roberto Franceschi, Mila Kalapurackal, Silvia Longhi, Michela Mariani, Marco Pitea, Andrea Secco, Daniele Tessaris, Francesco Vierucci, Malgorzata Wasniewska, Giovanna Weber, Stefano Mora

**Affiliations:** ^1^Pediatric and Adolescent Endocrinology, Division of Pediatrics, Department of Obstetrics, Gynecology and Pediatrics, University Hospital, Pisa, Italy; ^2^Department of Clinical and Experimental Medicine, Section of Paediatrics, University of Pisa, Pisa, Italy; ^3^Department of Human Pathology of Adulthood and Childhood, University of Messina, Messina, Italy; ^4^Pediatric Unit, University Hospital “G. Martino”, Messina, Italy; ^5^Pediatric Unit, IRCCS Azienda Ospedaliero-Universitaria di Bologna, Bologna, Italy; ^6^Department of Medical and Surgical Sciences, University of Bologna, Bologna, Italy; ^7^Pediatric Unit, Department of Precision and Regenerative Medicine and Ionian Area, University “A. Moro” of Bari, Bari, Italy; ^8^Division of Pediatric Endocrinology, Department of Public Health and Pediatrics, University of Turin, Regina Margherita Children’s Hospital, Turin, Italy; ^9^Department of Pediatrics, IRCCS Istituto Giannina Gaslini, Genova, Italy; ^10^Department of Neuroscience, Rehabilitation, Ophthalmology, Genetics, Maternal and Child Health, University of Genova, Genova, Italy; ^11^Endocrinology and Diabetology Unit, Bambino Gesù Children Hospital, IRCCS, Rome, Italy; ^12^Department of Pediatrics, Santa Chiara Hospital of Trento, APSS, Trento, Italy; ^13^Department of Pediatrics, Hospital of Bolzano (SABES-ASDAA), Teaching Hospital of Paracelsus Medical University (PMU), Bolzano, Italy; ^14^Pediatric Endocrinology Unit, Department of Pediatrics, IRCCS Ospedale San Raffaele, Milan, Italy; ^15^Pediatric and Pediatric Emergency Unit, Children Hospital, Azienda Ospedaliera SS Antonio e Biagio e C. Arrigo, Alessandria, Italy; ^16^Department of Pediatrics, San Luca Hospital, Lucca, Italy; ^17^Laboratory of Pediatric Endocrinology, Department of Pediatrics, IRCCS Ospedale San Raffaele, Milan, Italy

**Keywords:** hereditary rickets, management, non-hereditary rickets, nutritional rickets, treatment

## Abstract

Rickets results from impaired mineralization of growing bone due to alterations in calcium and phosphate homeostasis. Clinical signs of rickets are related to the age of the patient, the duration of the disease, and the underlying disorder. The most common signs of rickets are swelling of the wrists, knees or ankles, bowing of the legs (knock-knees, outward bowing, or both) and inability to walk. However, clinical features alone cannot differentiate between the various forms of rickets. Rickets includes a heterogeneous group of acquired and inherited diseases. Nutritional rickets is due to a deficiency of vitamin D, dietary calcium or phosphate. Mutations in genes responsible for vitamin D metabolism or function, the production or breakdown of fibroblast growth factor 23, renal phosphate regulation, or bone mineralization can lead to the hereditary form of rickets. This position paper reviews the relevant literature and presents the expertise of the Bone and Mineral Metabolism Group of the Italian Society of Pediatric Endocrinology and Diabetology (SIEDP). The aim of this document is to provide practical guidance to specialists and healthcare professionals on the main criteria for diagnosis, treatment, and management of patients with rickets. The various forms of rickets are discussed, and detailed references for the discussion of each form are provided. Algorithms to guide the diagnostic approach and recommendations to manage patients with rare forms of hereditary rickets are proposed.

## 1 Introduction

Rickets is a skeletal disease characterized by deficient mineralization of growth plates and bone matrix (osteomalacia) associated with low concentrations of calcium and/or phosphate in the blood. Rickets includes a heterogeneous group of acquired and inherited diseases. Nutritional rickets is the most common form of rickets globally. On the other hand, the development of molecular genetic techniques increased identification of the inherited forms of rickets. The primary method for diagnosing rickets involves examining the patient’s medical history, conducting biochemical tests, and performing radiologic examinations. Genetic analyses are particularly important in cases where patients are suspected to have a genetic form of rickets despite the absence of a family history of the disease. Treatment and management of rickets should be targeted on the pathogenesis and both are strictly connected with the diagnosis.

The aim of this document is to provide practical guidance to specialists and healthcare professionals about the main criteria for diagnosis, treatment, and management of patients with rickets.

## 2 Methods

This position paper focuses on the main criteria for the diagnosis, treatment, and management of patients with rickets. Relevant studies were identified independently by three authors (G.B., S.M., and P.C.) from the Medline/PubMed database. This document is the result of a thorough discussion by the members of the Bone and Mineral Metabolism Group of the Italian Society of Pediatric Endocrinology and Diabetology (SIEDP) to standardize the procedures based on the relevant literature and on the expertise of the panel members. We did not grade the strength of our recommendations, but much information reported in this paper was obtained from studies that used this method.

The aim of the document was not to provide a literature review on rickets or to discuss in detail the various form of rickets. The intention was to provide stakeholders with selected data and suggest a correct approach for an early diagnosis of rickets. Some algorithms to facilitate the diagnosis are also presented. Moreover, some important aspects of the management of patients with different forms of rickets are proposed. This position paper presents a therapeutic approach to the various forms of rickets in light of recent guidelines, and a specific treatment for patients with X-linked hypophosphatemic rickets (XLH).

## 3 Classification of rickets

Rickets may be classified in various forms based on the primary biochemical alteration, such as hypocalcemia or hypophosphatemia, or subdivided into two main groups, such as nutritional and hereditary forms. Moreover, some rare forms of rickets may be associated with direct bone damage due to acquired or inherited disorders.

**Figure 1:**
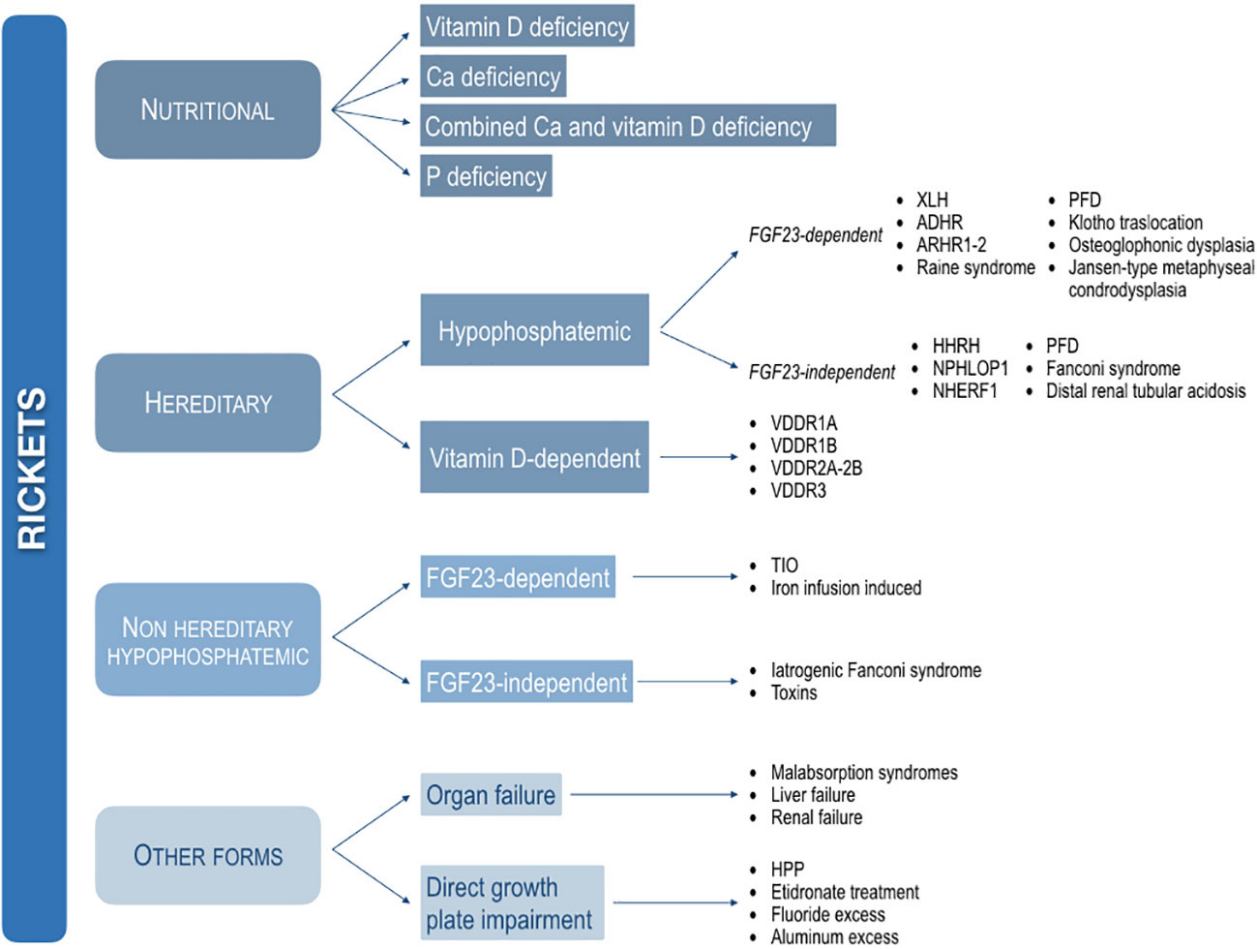
Classification of the main forms of rickets based on the pathogenesis. XLH, X-linked hypophosphatemic rickets; ADHR, autosomal dominant hypophosphatemic rickets; ARHR1, autosomal recessive hypophosphatemic rickets type 1; ARHR2, autosomal recessive hypophosphatemic rickets type 2; PFD, polyostotic fibrous dysplasia; HHRH, hypophosphatemic rickets with hypercalciuria; NPHLPO1, nephrolithiasis/osteoporosis, hypophosphatemic, 1. NHERF1, nephrolithiasis/osteoporosis, hypophosphatemic, 2; VDDR1A, vitamin D-dependent rickets type 1A; VDDR1B, vitamin D-dependent rickets type 1B; VDDR2A, vitamin D-dependent rickets type 2A; VDDR2B, vitamin D-dependent rickets type 2B; VDDR3, vitamin D-dependent rickets type 3; TIO, Tumor-induced osteomalacia; HPP, hypophosphatasia.

In this position paper, the various forms of rickets are subdivided into four major categories based on the main pathogenesis, such as nutritional, hereditary, non-hereditary hypophosphatemic, and other forms (**Figure 1**).

### 3.1 Nutritional rickets

Nutritional rickets is the most common form of rickets in developing countries, and it is frequently diagnosed in western countries among immigrant children, mainly from Africa and eastern Europe. Although it can rarely be caused by a phosphate-deficient diet, nutritional rickets is commonly the result of vitamin D deficiency due to insufficient UV exposure and/or insufficient dietary calcium intake, or a combination of both. This is particularly worrisome as nutritional rickets can profoundly affect the well-being of infants, children, and adolescents, with consequences that can persist into adulthood (1, 2).

In regions where calcium consumption is typically scant, with limited or absent dairy intake, dietary calcium deficiency stands as the primary culprit behind nutritional rickets among children beyond infancy (1, 3).

Dietary phosphate deficiency is a rare cause of nutritional rickets, but it is infrequent, because phosphate is abundant in natural foods. However, extreme starvation, a diet of elemental or hypoallergenic formula or parenteral nutrition, gastrointestinal surgery or short bowel syndrome, overuse of phosphate binders, or overly restrictive phosphate intake in managing chronic kidney disease may result in low phosphate intake (2, 4). Moreover, very low birthweight preterm infants are at high risk of developing nutritional rickets due to phosphate deficiency especially if they are breast-fed without phosphate supplements (2, 5).

### 3.2 Hereditary rickets

Hereditary rickets, caused by genetic mutations, may be subdivided into two main groups of disorders based on the association with impaired phosphate metabolism (hypophosphatemic rickets) or impaired vitamin D metabolism (vitamin D-dependent rickets) (**Figure 1**).

#### 3.2.1 Hypophosphatemic rickets

Hypophosphatemic rickets include some disorders that may be associated with overproduction of fibroblast growth factor 23 (FGF23), namely FGF23-dependent forms, and disorders associated with a primary renal defect with normal concentrations of circulating FGF23, namely FGF23-independent forms (**Figure 1**).

##### 3.2.1.1 FGF23-dependent hypophosphatemic rickets

X-linked hypophosphatemic rickets (XLH) is the commonest inherited form of rickets (6). Its prevalence is 1:20,000-60,000 (7, 8). XLH is due to mutation of *PHEX* gene encoding for phosphate regulating endopeptidase homolog X-linked, that regulates the expression of FGF23. The excessive production of FGF23 reduces tubular phosphate reabsorption and 1α-hydroxylase activity and stimulates renal 24-hydroxylase activity. Hypophosphatemia and a low or inappropriately normal serum 1,25-dihydroxyvitamin D (1,25(OH)_2_D) concentration in the setting of hypophosphatemia are the main biochemical findings of the patients with XLH (2, 9). Several other genes, such as *FGF23*, *DMP1*, and *ENPP1*, have a role in the synthesis, signaling, and regulation of FGF23. Mutations in these genes give rise to other rare variants of hypophosphatemic rickets, including autosomal dominant hypophosphatemic rickets (ADHR), autosomal recessive hypophosphatemic rickets type 1 (ARHR1) and autosomal recessive hypophosphatemic rickets type 2 (ARHR2), collectively constituting less than 20% of the cases (10).

Other rare forms of FGF23-dependent hypophosphatemic rickets include Raine syndrome due to mutation in the *FAM20C* gene (11), polyostotic fibrous dysplasia (PFD) due to gain-of-function mutation of the *GNAS* gene (12), hypophosphatemic rickets and hyperparathyroidism due to translocation of the *KLOTHO* promoter (13), osteoglophonic dysplasia caused by dominant activation variants in *FGFR1* (14), opsismodysplasia (15), and Jansen-type metaphyseal chondrodysplasia due to mutations in the parathyroid hormone 1 receptor gene (16, 17). Further details are reported by Ackah et al. (14) and Shore (18).

##### 3.2.1.2 FGF23-independent hypophosphatemic rickets

These disorders include some forms of hypophosphatemic rickets due to mutations in *SLC34A3* encoding for NaPi2C (hypophosphatemic rickets with hypercalciuria, HHRH), *SLC34A1* gene encoding for NaPi2a (nephrolithiasis/osteoporosis, hypophosphatemic 1, NPHLOP1), or mutation in *NHERF1* gene (nephrolithiasis/osteoporosis, hypophosphatemic, 2) (19, 20). Patients affected by Fanconi syndrome or distal renal tubular acidosis may develop hypophosphatemic rickets (2).

#### 3.2.2 Vitamin D-dependent rickets

Vitamin D-dependent rickets are caused by mutations affecting the enzymes involved in the activation or degradation of vitamin D or the action/expression of the vitamin D receptor (VDR). Vitamin D-dependent rickets type 1A (VDDR1A) is due to impaired synthesis of 1,25(OH)_2_D caused by a mutation in the enzyme (*CYP27B1*) encoding for renal 1α-hydroxylase. Vitamin D-dependent rickets type 1B (VDDR1B) is due to an impaired synthesis of 25-hydroxyvitamin D (25(OH)D) caused by a mutation in the enzyme (*CYP2R1*) encoding for hepatic 25-hydroxylase. Vitamin D-dependent rickets type 2 (VDDR2) is the result of impaired signaling of the VDR due to mutations in the *VDR* gene (VDDR2A) or the presence of a nuclear ribonucleoprotein that interferes with the VDR-DNA interaction (VDDR2B) (21). Vitamin D-dependent rickets type 3 (VDDR3) is due to increased inactivation of 1,25(OH)_2_D caused by a gain-of-function mutation in a gene encoding a vitamin D-degrading enzyme (*CYP3A4*) (21, 22). The most striking biochemical sign of these forms of rickets is severe hypocalcemia with secondary hyperparathyroidism.

### 3.3 Non-hereditary hypophosphatemic rickets

This group of hypophosphatemic rickets includes some heterogeneous acquired disorders causing phosphate wasting. They may be associated with increased production of FGF23 or iatrogenic causes.

#### 3.3.1 Acquired FGF23-dependent hypophosphatemic rickets

Tumor-induced osteomalacia (TIO) is due to the overproduction of FGF23 and other phosphaturic factors by benign slow-growing mesenchymal tumors (23, 24). TIO has rarely been described in children (25–30).

Iron infusion-induced reversible hypophosphatemic osteomalacia may occur in patients receiving intravenous iron preparations, especially with iron carboxymaltose or iron polymaltose, for treatment of anemia when oral iron is either ineffective or contraindicated (31). Iron infusion acutely impairs FGF23 cleavage, triggering transient increase in intact FGF23 and hypophosphatemia in association with an inappropriately low concentration of 1,25(OH)_2_D, similar to genetic diseases of primary FGF23 excess (14, 31).

#### 3.3.2 Acquired FGF23-independent hypophosphatemic rickets

Iatrogenic renal Fanconi syndromes with hypophosphatemia may be caused by some drugs, including valproate, cisplatin, ifosfamide, gentamycin, and excessive use of phosphate binders (32, 33) and by some toxins, such as heavy metals antibiotics, or antiretroviral and anticancer medications (14).

### 3.4 Other causes of rickets

Rickets may be a consequence of congenital or acquired chronic disorders affecting the organs involved in the activation of vitamin D. Liver failure and renal failure are the main causes of impaired vitamin D metabolism. Moreover, malabsorption syndromes, i.e., cystic fibrosis, inflammatory bowel diseases, coeliac disease, or extensive surgical intestinal resection, may affect the intestinal absorption of vitamin D. These heterogeneous forms of rickets are also associated with various degrees of reduced bone mineral density that may be assessed by dual energy X-ray absorptiometry (DXA) (2, 34, 35).

Some rare forms of rickets are caused by direct impairment of the process of mineralization at the growth plate. Protracted etidronate disodium (ethane 1-hydroxy-1, 1-diphosphonate) administration in patients with generalized arterial calcification has been associated with radiologic signs of rickets likely due to impaired growth plate mineralization (36, 37). Patients with the infantile form of hereditary hypophosphatasia (HPP) show radiological lesions that may resemble rickets. A poorly mineralized ribcage may be associated with respiratory insufficiency (38, 39).

A rare form of hypophosphatemic rickets with hypocalciuria has been reported after long-term treatment with aluminum-containing antacids (40, 41).

Furthermore, a particular form of rickets was observed in Indian children exposed to a high intake of endemic fluoride in the drinking water since their birth. The association with calcium deficiency was a trigger for the development of fluoride toxicity in bone. In calcium-deficient children, the toxic effects of fluoride manifested even at marginally high (> 2.5 mg/day) exposures to fluoride (42).

## 4 Clinical approach to rickets

Medical history, physical examination, and the biochemical results are the main findings to diagnose a form of rickets. The diagnosis of rickets should be confirmed by radiologic examination. The administration of vitamin D supplements, the evidence of relatives affected by rickets, the estimation of dietary calcium intake, and data on gestation and birth should be accurately investigated. Some clinical signs of rickets are more evident based on the age of the patient. Craniotabes (softening or thinning of the skull bones) may be the first sign of rickets in infants; it is detected by an inward collapse when applying pressure to the skull, typically followed by a snapping back after removing the pressure (43). In infants, other early signs of rickets are delayed fontanelle closure and frontal bossing (43, 44). In older children, swelling of the wrists, knees, or ankles and deformation of the legs such as knock-knees (genu valgum), or outward bowing (genu varum) with inability to walk are the main features (2, 44). Other skeletal signs of rickets are rachitic rosary, caused by swelling of the costochondral joints of the ribs, deformity of the soft rib cage, and bone pain (2, 44). Several non-osseous features may be associated with rickets: failure to thrive, delayed motor development, convulsions (due to hypocalcemia), muscular hypotonia, dilated cardiomyopathy, anemia, lethargy, irritability, delayed tooth eruption with altered enamel, and predisposition to respiratory infections (1, 43–48). HPP is characterized by diverse phenotypes that contribute to the diagnostic difficulties. In particular, neurological and dental involvements advocate for a multidisciplinary approach in the diagnosis of HPP (49).

Physical examination cannot differentiate children with active rickets from children recovering from rickets, and between the various forms of rickets (43). In addition, if abnormal bone features are detected clinically, the disease is already well established. Relying only on clinical signs may lead to overestimation of the prevalence of active rickets and lead to unnecessary treatment. The main skeletal signs of rickets are summarized in **Table 1**.

**Table 1:** Main skeletal and dental-periodontal signs in patients with rickets.

Cranium	Thorax and pelvis	Limbs	Total body and spine	Teeth and periodontium
• Frontal bossing • Craniosynostosis • Scaphocephaly • Occipital “bullet deformity” • Delayed anterior fontanel closure • Craniotabes^a^ • Mid facial hypoplasia^b^	• Costo-chondral junction enlargement (rachitic rosary)^a^ • Harrison sulcus^a^ • Costal pathological fractures^a^ • Pigeon chest • Chest wall asymmetry • Depressed ribs • Narrowed pelvic outlet	• Widened wrists, knees, and ankles • Genu-varum • Genu-valgum • Combined genu-varum/valgum • Short humerus^b^ • Short femur^b^ • Tibial torsion^c^ • Coxa-vara	• Stunted growth • “Taylorwise” posture^a^ • Disproportionate short stature (short limbs)^b^ • Spinal curvature • Kyphosis	• Multiple dental decay^a^ • Dyschromic enamel • Enamel hypoplasia • Delayed dentition • Abscesses with gingival fistulae^d^

^a^Mainly in patients with nutritional vitamin D deficiency rickets or vitamin D-dependent rickets; ^b^mainly in patients with XLH; ^c^intoeing or extoeing; ^d^typical of patients with XLH: mainly in incisors and canines, without evidence of trauma or dental decay.

## 5 Biochemical features of the various forms of rickets

Serum calcium, phosphate, alkaline phosphatase, parathyroid hormone (PTH), and vitamin D metabolites are crucial biochemical parameters for the diagnosis of the various forms of rickets. Increased alkaline phosphatase activity is evident in all forms of rickets, except HPP (2, 4). Serum concentrations of phosphate and alkaline phosphatase activity should be interpreted according to appropriate age-specific reference values (49, 50).

Hypocalcemia is a main biochemical finding in patients with severe nutritional vitamin D deficiency rickets and in patients with vitamin D-dependent rickets. Hypophosphatemia (with normal serum calcium concentrations) is a specific feature in patients with hypophosphatemic rickets. However, hypophosphatemia may be also evident (usually associated with hypocalcemia) in patients with severe nutritional vitamin D deficiency rickets as a consequence of secondary hyperparathyroidism (2, 4, 35). It has been demonstrated that hypophosphatemia is the common denominator of all rickets affecting the process of apoptosis in the hypertrophic cells in the growth plate. In the absence of apoptosis, the hypertrophic cells accumulate in the growth plate and form the rachitic bone (51).

Renal tubular reabsorption of phosphate can be assessed by calculating the ratio of the tubular maximum reabsorption of phosphate to the glomerular filtration rate (TmP/GFR) (52). A reduced TmP/GFR ratio in patients with nutritional vitamin D deficiency rickets or vitamin D-dependent rickets reflects secondary hyperparathyroidism (2). The reduced TmP/GFR ratio found in patients with hypophosphatemic rickets is due to an overproduction or reduced degradation of fibroblast growth factor 23 (FGF23) (4, 20, 53).

Hypercalciuria may be an important diagnostic criterion in patients with HHRH (2, 4, 20, 35).

Secondary hyperparathyroidism is a major biochemical feature in patients with hypocalcemic forms of rickets. Serum PTH concentrations are normal or only slightly increased in patients with hypophosphatemic rickets, except in rare instances, such as in patients with HHRH who showed low normal o reduced serum PTH concentrations (2, 4, 20, 53).

There is strong evidence that nutritional vitamin D deficiency rickets develops with serum 25(OH)D concentrations <30 nmol/L (<12 ng/mL) (1), but it may also be associated with serum 25(OH)D concentrations >30 nmol/L (>12 ng/mL) (1, 54, 55). Furthermore, most children with vitamin D deficiency are asymptomatic (2, 56–58), and a normal calcium intake could be able to maintain bone integrity (1). Some evidence suggested that a 25(OH)D concentration of 30 - 34 nmol/L (12 - 13.6 ng/mL) may be the critical cutoff value below which nutritional vitamin D deficiency rickets is more likely to occur (1, 59). A recent systematic review and multi-level meta-analyses by odds, sensitivities and specificities for nutritional rickets at different serum 25(OH)D thresholds suggested a minimal risk threshold of around 28 nmol/L (11 ng/ml) for children with adequate calcium intakes and 40 nmol/L (16 ng/ml) for children with low calcium intakes (60). Indeed, the total concentration of 25(OH)D and its circulating components may be influenced by factors such as the severity and duration of reduced 25(OH)D levels, habitual intake and bioavailability of dietary calcium, the rate of bone growth and mineralization, as well as genetic polymorphisms (2, 55, 61).

Reduced serum 25(OH)D concentrations are observed in patients with vitamin D hydroxylation-deficient rickets type 1B (62) and VDDR3 (22); conversely, patients with VDDR1A and VDDR2A or VDDR2B have normal or increased serum 25(OH)D concentrations (21).

Serum 1,25(OH)_2_D concentrations vary with the time of diagnosis in nutritional vitamin D deficiency rickets. They are generally increased at the early stages of the disease to compensate for hypocalcemia and secondary hyperparathyroidism, whereas they are decreased at the late stages of the disease due to substrate depletion (48, 51). Therefore, measurement of 1,25(OH)_2_D is not useful in patients with nutritional vitamin D deficiency rickets. In patients with hypophosphatemic rickets, serum 1,25(OH)_2_D concentrations may be reduced, but in most patients, they are inappropriately normal in the setting of hypophosphatemia (2, 4, 35, 53). Serum 1,25(OH)_2_D concentrations are increased in patients with VDDR2A, VDDR2B, nutritional calcium deficiency rickets, X-linked recessive hypophosphatemic rickets, HHRH or NPHLPO1 (2, 4, 20, 35).

FGF23 concentrations are increased in FGF23-dependent hypophosphatemic rickets (2, 4, 20, 35, 50); therefore, these conditions can be distinguished from FGF23-independent hypophosphatemic disorders by measuring the FGF23 concentration. Hypophosphatemia associated with intact FGF23 concentration greater than 40 pg/ml may be a crucial marker for the early diagnosis of XLH in pediatric patients (50). Assessment of FGF23 is also indicated in the diagnosis of patients with suspected TIO. Furthermore, FGF23 is the most critical biochemical marker for distinguishing nutritional vitamin D deficiency rickets from hypophosphatemic rickets, mainly in patients with an overlap of serum 25(OH)D concentrations (63). Moreover, increased FGF23 concentration is found in patients with calcium deficiency rickets (64).

Hypercalcemia and hyperphosphatemia, in addition to reduced alkaline phosphatase activity, are useful parameters for the differential diagnosis of the various forms of rickets (38, 39).

Biochemical features of the main forms of rickets are shown in **Table 2**.

**Table 2:** Biochemical findings in patients with different types of rickets or rickets-like disorders.

Disorder	sCa	sP	ALP	PTH	25(OH)D	1,25(OH)_2_D	FGF23	uCa	Tmp
Nutritional rickets									
Nutritional vitamin D deficiency rickets	N, ↓	↓	↑	↑	↓	↑, N, ↓	N	↓	↓
Nutritional calcium deficiency rickets	N, ↓	↓	↑	↑	N, ↓	↑	N, ↑	↓	↓
Nutritional phosphate deficiency	N, ↑	↓	↑	N, ↓	N	N, ↑	N, ↓	N, ↑^a^	N, ↓^b^
Hereditary FGF23-dependent hypophosphatemia									
XLH, ADHR, ARHR1, ARHR2	N	↓	↑	N, ↑^c^	N	N^d^, ↓	N, ↑	N, ↓	↓
Raine syndrome	N, ↓^e^	N, ↓ ^e^	↑	N, ↑	N	N, ↓	N, ↑	N	↓
PFD	N, ↑	N, ↓	↑	N, ↑	N	N, ↓	N, ↑	N	N, ↓
Hypophosphatemic rickets and hyperparathyroidism	N, ↑	↓	↑	↑	N	N^d^, ↓	↑	N, ↑	↓
Osteoglophonic dysplasia	N	↓	N, ↑	N	N	N^d^, ↓	N, ↑	N	↓
Opsismodysplasia	N	↓	N, ↑	N	N	N^d^, ↓	N, ↑	N	↓
Jansen-type metaphyseal chondrodysplasia	N, ↑	↓	↑	N, ↓	N	N, ↑	↑	N, ↑	↓
Hereditary FGF23-independent hypophosphatemia									
HHRH	N	↓	↑	N, ↓	N	N, ↑	N, ↓	↑	↓
NPHLPO1	N	↓	↑	variable	N	↑	↓	↑	↓
NHERF1	N	↓	?	N	N	N, ↑	N	↑	↓
X-linked recessive hypophosphatemic rickets	N	↓	↑	variable	N	↑	?	N, ↑	↓
Fanconi syndrome or distal renal tubular acidosis	↑, N, ↓	N, ↓	↑	N, ↑^e^	N	N, ↓	N, ↑^f^	N, ↑	N, ↓
Linear sebaceous nevus syndrome	N, ↓	↓	↑	N, ↑^c^	N	N^d^, ↓	N, ↑	N, ↓	↓
Vitamin D-dependent rickets									
VDDR1A	↓	↓	↑	↑	N	↓	N, ↓	↓	↓
VDDR1B	↓	↓	↑	↑	↓	↓	N	↓	↓
VDDR2A-2B	↓	↓	↑	↑	N	↑	N, ↓	↓	↓
VDDR3	↓	↓	↑	↑	↓	↓	?	↓	↓
Acquired FGF23-dependent hypophosphatemia									
Tumor-induced osteomalacia	N, ↓	↓	↑	N, ↑^c^	N	N^d^, ↓	N, ↑	N, ↓	↓
Ferric carboxymaltose i.v. infusion	↓	↓	↑	↑	N	↓	↑^f^, ↓^g^	?	↓
Acquired FGF23-independent hypophosphatemia									
Iatrogenic proximal Fanconi syndromes	N	↓	↑	variable	N	↑	↓	variable	↓
Other causes of rickets									
Malabsorption, liver insufficiency/failure, renal insufficiency/failure	N, ↓	↑, N, ↓	↑	N, ↑	N, ↓	variable	↑^h^	variable	↓ ^i^
Etidronate treatment	N	↓	↑	N	N	↑	?	N	↓
HPP	N, ↑	↑	↓	N, ↓	N	N, ↓	N	↑	↑

sCa, serum calcium; sP, serum phosphate; ALP, alkaline phosphatase; PTH, parathyroid hormone; 25(OH)D, 25-hydroxyvitamin D; 1,25(OH)_2_D, 1,25-dihydroxyvitamin D; FGF23, fibroblast growth factor 23; uCa, urinary calcium, TmP, renal tubular reabsorption of phosphate.

XLH, X-linked hypophosphatemic rickets; ADHR, autosomal dominant hypophosphatemic rickets; ARHR1, autosomal recessive hypophosphatemic rickets type 1; ARHR2, autosomal recessive hypophosphatemic rickets type 2; PFD, polyostotic fibrous dysplasia; HHRH, hypophosphatemic rickets with hypercalciuria; NPHLPO1, nephrolithiasis/osteoporosis, hypophosphatemic, 1. NHERF1, nephrolithiasis/osteoporosis, hypophosphatemic, 2; VDDR1A, vitamin D-dependent rickets type 1A; VDDR1B, vitamin D-dependent rickets type 1B; VDDR2A, vitamin D-dependent rickets type 2A; VDDR2B, vitamin D-dependent rickets type 2B; VDDR3, vitamin D-dependent rickets type 3; HPP, hypophosphatasia.

↑: increased concentration; ↓: decreased concentration; N: normal concentration;?: not known.

Nutrional phosphate deficiency: biochemical findings are referred to dietary phosphate deficiency in very low birthweight infants.

^a^Normal after restoration of P intake, but falsely increased before restoration.

^b^Normal after restoration of P intake, but falsely reduced before restoration. Urinary phosphate excretion is very low or undetectable before restoration of P intake.

^c^It may be moderately above the upper limit of normal.

^d^decreased relative to the serum phosphate concentration.

^e^depending on the stage of chronic kidney disease.

^f^Intact FGF23.

^g^C-terminal FGF23.

^h^in patients with renal insufficiency/failure.

^i^in patients with secondary hyperparathyroidism or excessive production of FGF23 due to renal insufficiency/failure.

## 6 Radiological assessment of rickets: diagnosis and follow-up

Plain radiography is the primary imaging tool for the assessment of rickets; the earliest signs are osteopenia and cortical thinning of long bones (48, 65). The initial assessment of a child suspected of having rickets should involve radiographs of skeletal sites where bones are undergoing the most rapid growth. These sites may differ depending on the child’s age.

A radiological examination of the chest may be useful to assess rickets in neonates, mainly in preterm infants, and in infants in the first year of life (65, 66). Insufficient mineralization of the metaphyseal-physeal region can be observed in the proximal humerus and anterior rib ends (rachitic rosary). In most children, radiological signs of rickets are evident at the wrists and knees, where rapid growth occurs. The distal ulna is the best site to demonstrate early changes of rickets, mostly in young children (67). The classical rachitic changes are the widening and irregularity of all the physes, with metaphyseal widening, splaying, cupping, and fraying. Moreover, a coarse trabecular pattern may be observed in the metaphysis (48, 65). Epiphyseal ossification centers become blurred or apparent (65). Cranial deformities and bowing of the legs are the typical signs of osteomalacia due to unmineralized osteoid (66).

Pathologic fractures (including rib fractures) are rare except in very preterm babies and are very uncommon beyond age 6 months corrected postnatal age (66). The occurrence of fractures is usually evident in children with a severe form of rickets (68). Fractures of long bones may occur occasionally in children affected by calciopenic and phosphopenic (excluding XLH) forms of rickets in association with radiographic signs of osteopenia and the evidence of typical metaphyseal rachitic lesions (2).

Moreover, by radiologic examination is possible to estimate the severity of rickets by using the Rickets Severity Score (RSS). This score is determined based on the degree of metaphyseal fraying, concavity, and the proportion of the growth plate affected at the wrists, knees, and ankles (69, 70). The RSS employs a 10-point scale, with 10 indicating the utmost severity of rickets, and 0 denoting the absence of any radiographic alterations. The radiographic response after treatment of nutritional vitamin D deficiency rickets (69) and XLH (70) can be estimated by the RSS, which correlates with serum alkaline phosphatase activity.

The Radiographic Global Impression of Change (RGI-C) represents a complementary assessment of RSS. A change score is assigned based on differences in the appearance of rickets on pairs of radiographs compared side by side. The RGI-C has been validated to evaluate skeletal changes in patients with HPP (71). Moreover, Lim et al. (72) demonstrated that RGI-C is a reliable, valid, and sensitive tool in patients with XLH, and complementary to the RSS.

**Figure 2:**
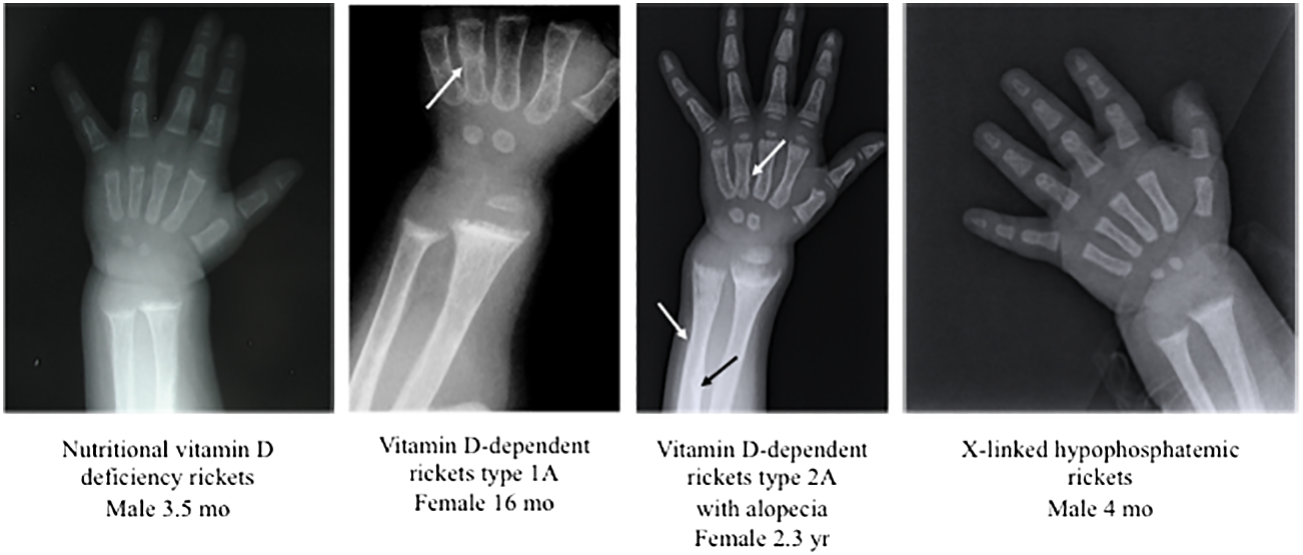
Radiographic features at the wrist and at the hand in patients with nutritional vitamin D deficiency rickets and some forms of hereditary rickets. In patients with hereditary rickets, the diagnosis was confirmed by genetic analyses. All patients showed widening and fraying of the epiphyseal plate and metaphyseal concavity of the ulna. The white and black arrows showed fractures.

Radiologic examination is also useful to confirm the diagnosis of rickets suspected by clinical signs and biochemical data, but it does not indicate the pathogenesis, because the radiological signs of rickets are similar in patients with nutritional rickets, hypophosphatemic rickets, and vitamin D-dependent rickets (**Figure 2**).

Finally, radiological examination is useful to examine the effect of treatment with vitamin D in patients with nutritional vitamin D deficiency rickets showing the appearance of the zone of provisional calcification at the ends of the metaphyses that is usually seen within 3-4 weeks of treatment (67).

## 7 Algorithms for the diagnosis of rickets

**Figure 3:**
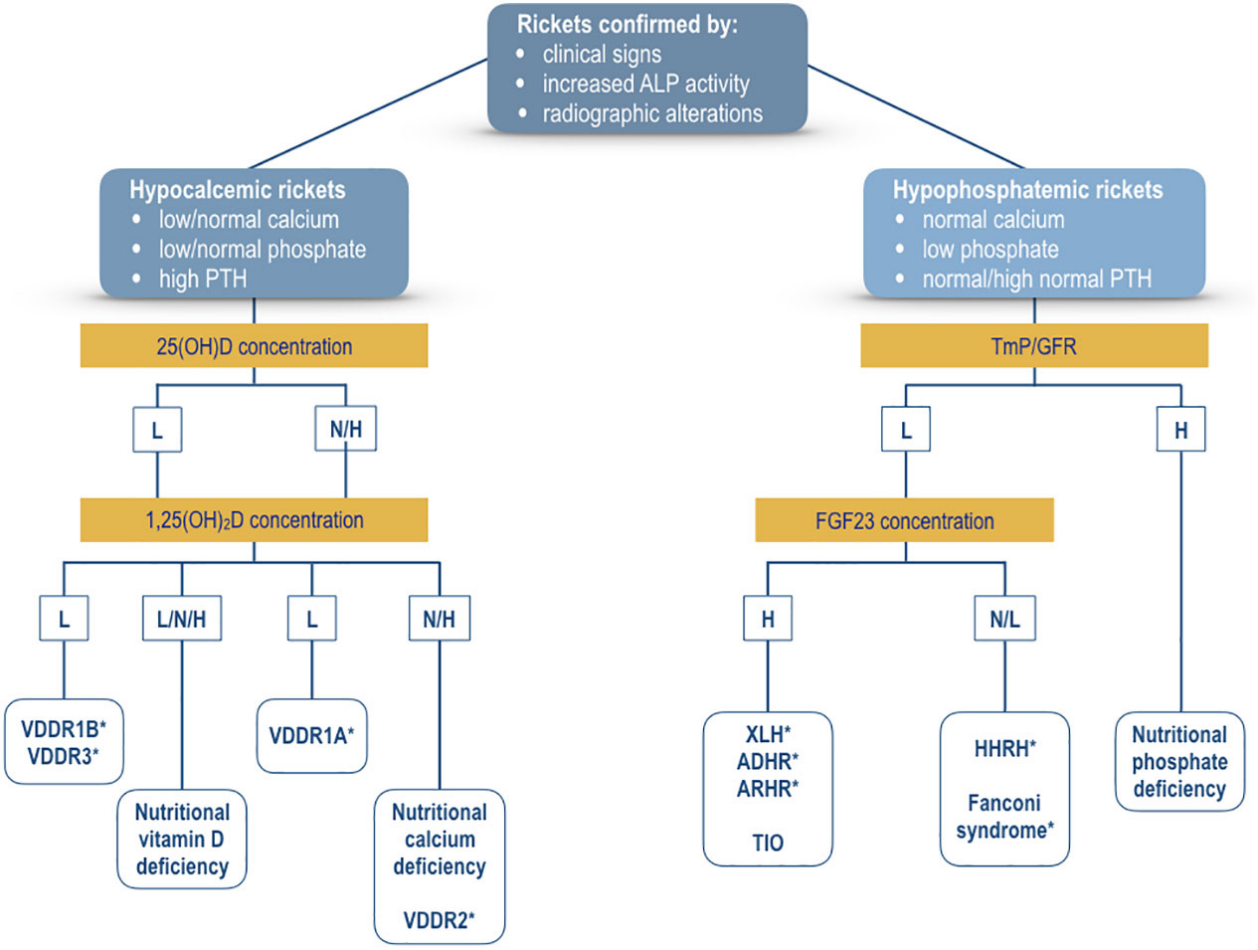
Algorithm for the evaluation of a patient with confirmed rickets by clinical signs, increased alkaline phosphatase activity, and radiographic lesions of rickets. The primary differential diagnosis is based on the presence of hypocalcemia (hypocalcemic rickets) or hypophosphatemia (hypophosphatemic rickets). Measurement of serum concentrations of 25(OH)D and 1,25(OH)_2_D may indicate the biochemical diagnosis of the various forms of hypocalcemic rickets. Measurement of TmP/GFR values represents the first step for the selection of the patients with hypophosphatemic rickets. Thereafter, measurement of FGF23 concentration may suggest the biochemical diagnosis of the various forms of hypophosphatemic rickets. For additional detais see text. XLH, X-linked hypophosphatemic rickets; ADHR, autosomal dominant hypophosphatemic rickets; ARHR1, autosomal recessive hypophosphatemic rickets type 1; ARHR2, autosomal recessive hypophosphatemic rickets type 2; HHRH, hypophosphatemic rickets with hypercalciuria; VDDR1A, vitamin D-dependent rickets type 1A; VDDR1B, vitamin D-dependent rickets type 1B; VDDR2A, vitamin D-dependent rickets type 2A; VDDR2B, vitamin D-dependent rickets type 2B; VDDR3, vitamin D-dependent rickets type 3; TIO, Tumor-induced osteomalacia. *Genetic testing is important to confirm the suspected diagnosis. L, low values; N, normal values; H, high values.

**Figure 3** shows a management algorithm based on the biochemical parameters for the evaluation of patients with rickets confirmed by the evidence of clinical signs and the typical radiologic lesions of rickets and associated with increased serum alkaline phosphatase activity. The algorithm is primarily based on the pathogenesis of hypocalcemia and hypophosphatemia; serum concentrations of calcium, phosphate, and PTH represent the key biochemical parameters to differentiate hypocalcemic from hypophosphatemic form of rickets. Hypocalcemic forms of rickets are usually characterized by hypocalcemia, a low or normal serum phosphate concentration, and increased PTH values; whereas, hypophosphatemia with normocalcemia and normal PTH concentrations are the main biochemical signs of most of the hypophosphatemic forms of rickets.

The measurement of serum concentrations of vitamin D metabolites may indicate the pathogenesis of rickets in patients with hypocalcemic forms. Reduced serum values of 25(OH)D and 1,25(OH)_2_D suggest the diagnosis of VDDR1B or VDDR3, whereas normal or increased serum 25(OH)D values associated with low 1,25(OH)_2_D may be diagnostic for VDDR1A. A normal or increased serum 25(OH)D concentration combined with a normal or increased serum 1,25(OH)_2_D concentration may characterize two conditions, nutritional calcium deficiency or VDDR2.

The measurement of FGF23 may be useful to identify the FGF23-mediated from the non-FGF23-mediated hypophosphatemic disorders. High FGF23 concentrations are evident in both hereditary and acquired forms of hypophosphatemic rickets such as TIO. Hypophosphatemia associated with normal or low FGF23 concentrations suggests the diagnosis of a form of Fanconi syndrome or HHRH. Patients with HHRH usually show hypercalciuria. Hypophosphatemia with high TmP/GFR values may indicate the diagnosis of nutritional phosphate deficiency rickets.

## 8 Genetics

Whenever the clinical, biochemical, and radiologic evidence indicates that the cause of rickets might be of genetic origin, specific molecular testing is necessary. **Table 3** summarizes the genes involved in the genesis of genetically determined rickets or hypophosphatemia.

**Table 3:** Genes involved in the genesis of genetically determined rickets or hypophosphatemia.

Disorder	Gene	OMIMM #
XLH	*PHEX*	307800
ADHR	*FGF23*	193100
ARHR1	*DMP1*	241520
ARHR2	*ENPP1*	613312
Raine syndrome	*FAM20C*	259775
PFD	*GNAS*	174800
HHRH	*SLC34A3*	241530
Hypophosphatemic rickets and hyperparathyroidism	*13q13.1*	612089
Osteoglophonic dysplasia	*FGFR1*	166250
Opsismodysplasia	*INPPL1*	258480
Jansen-type metaphyseal chondrodysplasia	*PTH1R*	156400
NPHLOP1	*SLC34A1*	612286
NHERF1	*SLC9A3*	604990
X-linked recessive hypophosphatemic rickets	*CLCN5*	300554
VDDR1A	*CYP27B1*	264700
VDDR1B	*CYP2R1*	600081
VDDR2A	*VDR*	277440
VDDR2B	*VDR*	600785
VDDR3	*CYP3A4*	619073

XLH, X-linked hypophosphatemic rickets; ADHR, autosomal dominant hypophosphatemic rickets; ARHR1, autosomal recessive hypophosphatemic rickets type 1; ARHR2, autosomal recessive hypophosphatemic rickets type 2; PFD, polyostotic fibrous dysplasia; HHRH, hypophosphatemic rickets with hypercalciuria; NPHLPO1, nephrolithiasis/osteoporosis, hypophosphatemic, 1. NHERF1, nephrolithiasis/osteoporosis, hypophosphatemic, 2; VDDR1A, vitamin D-dependent rickets type 1A; VDDR1B, vitamin D-dependent rickets type 1B; VDDR2A, vitamin D-dependent rickets type 2A; VDDR2B, vitamin D-dependent rickets type 2B; VDDR3, vitamin D-dependent rickets type 3.

The analysis of the *PHEX* gene in the suspect of XLH should be performed, bearing in mind the extensive heterogeneity of the pathogenic variants. These have been found in all exons and in the non-coding regions. Numerous are the insertions and deletions that have also been reported (73, 74).

## 9 Treatment and management of nutritional rickets

### 9.1 Nutritional vitamin D deficiency rickets

Vitamin D supplements may be administered as daily therapy or as a single bolus; the suggested doses are reported in **Table 4**. Alternatively, a bolus of 100,000 IU every 20 days for 3-4 times may be effective in patients with poor compliance with the treatment (75). Administration of a single-day high-dose of vitamin D therapy (stoss therapy), with doses reaching up to 600,000 IU, has been proposed. However, hypercalcemia may occur (76, 77). Therefore, the use of high and repeated doses of vitamin D is not recommended. Daily treatment with vitamin D should be continued for 3-4 months (1, 2, 35, 48, 75, 78), but the duration of treatment should be individualized. Normalization of skeletal lesions is usually reached during this time. If radiographic recovery remains incomplete or serum biochemistry hasn’t normalized, vitamin D treatment should be extended (79). Inadequate adherence to the treatment regimen may lead to failure or delayed healing of rickets. In this occurrence, vitamin D stoss therapy by a single intramuscular injection ranging from 50,000 IU for children aged 3 months and older, up to 300,000 IU for individuals over 12 years may be recommended (1).

**Table 4:** Vitamin D treatment in patients with nutritional vitamin D deficiency rickets.

Age	Daily dose for 3 months, IU	Single dose, IU	Maintenance daily dose, IU
< 3 months	2000	–	400
3-12 months	2000	50,000	400
> 12 months to 12 years	3000-6000	150,000	600
> 12 years	6000	300,000	600

Response to treatment based on clinical, biochemical, and radiologic assessments, should be performed after 3 months of vitamin D administration; some patients may require further treatment. A daily calcium intake of at least 500 mg should be ensured, either as a dietary intake or supplements. For conversion from IU to μg, divide by 40.

Adapted from Munns et al. (1).

Some studies showed that in increasing serum 25(OH)D concentration the daily administration of vitamin D_3_ was similar to vitamin D_2_, whereas, vitamin D_3_ was more effective than vitamin D_2_ when administered as bolus (80). Moreover, there is no indication for the use of vitamin D metabolites for the treatment of patients with nutritional vitamin D deficiency rickets, because they do not restore vitamin D status and hypercalcemia may occur (66, 67, 81).

Patients experiencing symptomatic hypocalcemia, such as tetany, convulsions, or dilated cardiomyopathy, should receive intravenous calcium gluconate until serum calcium levels are normalized (35, 75, 78). Once normocalcemia is achieved, transitioning to oral calcium supplementation is appropriate. A routine oral calcium supplementation of 500 mg/day, obtained through dietary intake or supplements, should be implemented concurrently with vitamin D, irrespective of age or weight. Duration of calcium supplementation is variable and is related to the timing of normalization of serum calcium concentration (1). Alternatively, 30-75 mg/kg/day elemental calcium in 3 divided doses may be administered over 2-4 weeks. Calcium supplements are important to avoid hypocalcemia as the concentration of PTH normalizes (“hungry-bone” syndrome), particularly in patients treated with stoss therapy (48). Doses of intravenous and oral calcium supplements are reported in **Table 5**.

**Table 5:** Calcium treatment in patients with symptomatic or asymptomatic hypocalcemia associated with rickets.

Condition	Calcium salts	Doses, mg/kg^a^ (mL)	Mode of administration
Symptomatic acute hypocalcemia	Gluconate 10%^b^	5-20 (0.5-2.0)	Intravenous slowly, over 10-15 min to avoid bradycardia^c^; diluted in 0.9% sodium chloride or 5% dextrose
Asymptomatic hypocalcemia or normocalcemia	Carbonate, citrate	30-75 5 years old: 500 mg/day^d^ 10 years old: 1000 mg/day^d^	Oral route, divided into 2-3 doses daily

^a^As elemental calcium. Calcium carbonate is 40% elemental calcium; calcium citrate is 21% elemental calcium.

^b^One ampoule (10 mL) contains about 90 mg of elemental calcium.

^c^Electrocardiographic monitoring is strongly recommended.

^d^Suggested by Lambert AS and Linglart A (35).

Monitoring of treatment with vitamin D and calcium supplements varies with the severity of rickets and response to therapy. Normalization of serum calcium and phosphate concentrations usually occurs within 3 weeks (67, 75), but it may also be evident after only 6-10 days of treatment (48). Serum PTH concentrations usually fall within the normal range as normocalcemia is restored (75, 81). Serum 25(OH)D concentrations increase rapidly and normal values may be reached after 4-6 weeks (75). Serum 1,25(OH)_2_D concentrations increase rapidly with treatment and remain elevated for up to 10 weeks (81). Alkaline phosphatase activity declines progressively but it may remain increased for several months (3-6 months) depending on the severity of the vitamin D deficiency (48, 82). Moreover, alkaline phosphatase activity is associated with the recovery of skeletal rachitic lesions, suggesting that it is a reliable and economic biochemical marker for monitoring the effectiveness of treatment in the clinical setting (82).

In order to prevent the resurgence of nutritional vitamin D deficiency rickets vitamin D supplementation should be continued. Following the resolution of rickets, at least 400 IU/day before the age of 12 months and 600 IU/day of vitamin D are recommended (1, 67, 78).

### 9.2 Nutritional calcium deficiency rickets

A randomized controlled study in children with nutritional calcium-deficiency rickets showed that radiographic healing of the rachitic lesions was obtained by daily administration of with 500 mg, 1000 mg, or 2000 mg of elemental calcium salts. However, daily supplementation with 1000 mg and 2000 mg was more effective than 500 mg. The treatment with 2000 mg was similar to 1000 mg of supplemental calcium (83). Therefore, it is recommended that 1000 mg of elemental calcium daily (subdivided into 2-3 doses) should be used in children with nutritional calcium deficiency rickets, until healing is complete; in some children, it may take more than 24 weeks (84). The rate of healing of rickets may be improved by vitamin D supplements (50,000 IU every 4 weeks) (84). Radiologic healing of the growth plate usually occurred after 3-6 months of calcium administration, even though the clinical signs of rickets required a longer time to resolve than biochemical and radiologic alterations (3).

### 9.3 Nutritional phosphate deficiency rickets

Management of dietary phosphate deficiency is based primarily on adequate dietary supplements. Oral or parenteral phosphate salts are usually administered in patients with impaired phosphate absorption after extensive gastrointestinal surgery, short bowel syndrome, or severe gastrointestinal disorders (78).

Biochemical parameters, including relative hypocalcemia or hypophosphatemia, are the main factors for the management of metabolic bone disease of prematurity. Measurement of PTH has a pivotal role for the treatment, because increased PTH occurs in calcium deficiency, while it is normal or reduced in phosphate deficiency (85, 86). Human milk fortifiers and special preterm formulas are usually indicated to improve the needs for growth in very low birthweight infants. Phosphate supplementation should be considered for serum phosphate concentrations <4 mg/dL (1 mmol/L), but it can be also indicated if values fall below 5.5 mg/dL (1.3 mmol/L), especially if associated with increased alkaline phosphatase activity, to improve bone mineralization and to prevent hypercalciuria (87). The recommended daily oral intake of calcium and phosphate varies between 150-220 mg/kg/day and 75-140 mg/kg/day through enteral feeds, respectively (87–91); an enteral calcium to phosphate intake ratio at 1.5:1 to 1.7:1 on mg-to-mg basis is proposed (86, 87). For parental nutrition, calcium 75-100 mg/kg/day and phosphate 50-80 mg/kg/day are recommended (85, 86, 90, 91).

In infants unable to tolerate human milk fortifier or preterm formula, elemental minerals may be added by oral route. Supplementation usually starts at 20 mg/kg/day of elemental calcium and 10-20 mg/kg/day of elemental phosphorus. It is increased, as tolerated, to a maximum of 70-80 mg/kg/day of elemental calcium and 40-50 mg/kg/day of elemental phosphorus (87, 91). Serum PTH concentrations and alkaline phosphatase activity should be measured every 1-2 weekly for adjusting phosphate and calcium to phosphate ratios (86).

Vitamin D supplements in preterm infants differ between United States and Europe recommendations. The European Society for Paediatric Gastroenterology, Hepatology and Nutrition (ESPGHAN) recommends a dose of 800-1000 IU vitamin D daily, whereas 200 IU/day is recommended in the United States for infants weighing less than 1500 g and 400 IU/day for infants weighing more than 1500 g (87, 88, 91, 92). A lower calcium intake may benefit from a higher vitamin D intake as suggested by ESPGHAN recommendations, whereas a higher calcium intake may be preferred in association with a lower vitamin D intake as indicated in the United States (91, 92).

## 10 Treatment and management of vitamin D-dependent rickets

### 10.1 VDDR1A

Conventional treatment for patients with VDDR1A is based on the association of a vitamin D active metabolite and calcium supplements. Patients must be treated lifelong with physiologic doses of 1,25(OH)_2_D given twice daily due to its short half-life (21). Alternatively, alfacalcidol may be administered once a day due to its longer half-life. Alfacalcidol treatment by drop formulation may be useful in affected infants. The dosage of both calcitriol and alfacalcidol should be titrated according to serum calcium concentrations. The high calcium demands of the unmineralized skeleton occurring in the first months of treatment may require 2-5 times higher dosages of active vitamin D metabolites than during maintenance treatment (21, 93). The maintenance doses of active vitamin D metabolites and calcium supplements after normalization of serum calcium and PTH concentration are reported in **Table 6**.

**Table 6:** Maintenance treatment in patients with vitamin D-dependent rickets.

Drug	VDDR1A	VDDR1B	VDDR2	VDDR3
Vitamin D	NI	Heterozygous: 5,000-10,000 IU/day Homozygous: 600,000 every 3 months	LI	50,000/day
Calcifediol	NI	15-50 μg/day	20-200 μg/day^a^	?
Alfacalcidol	10-100 ng/kg/day 0.5-3 μg/day	0.5-3 μg/day	10-400 ng/kg/day^b^ 5-60 μg/day^b^	?
Calcitriol	10-100 ng/kg/day 0.3-2 μg/day	0.3-2 μg/day	10-400 ng/kg/day^b^ 5-60 μg/day^b^	?
Calcium salts (by oral route)	0.5-3 g/day	0.5-2 g/day	3-5 g/day 400-1400 mg/m^2^/day^c^	?

NI, not indicated.

LI, little indicated.

^a^only some patients without alopecia may respond.

^b^some patients do not respond despite maximal doses.

^c^intravenous administration for many years.

Adapted and modified from Levine MA (21).

Response to calcitriol or alfacalcidol is usually rapid, with healing of rickets and normalization of the biochemical parameters within 7-9 weeks (94). Long-term compliance with the treatment is important to maintain normal calcium and PTH concentrations. Serum concentrations of calcium, phosphate, and PTH, and urinary calcium excretion should be measured regularly during the maintenance treatment (at least every 4-6 months). However, time intervals may be reduced if hypocalcemia persists during the first weeks of treatment. Serum calcium concentration should be maintained in the low-normal range to keep PTH secretion below the upper limit of normal (95). Calcium excretion should be maintained below the normal limit for age and weight. Renal ultrasound examination should be performed every 1-2 years to monitor for renal calcification, and more frequently if there is evidence of hypercalciuria (21). However, based on personal expertise renal ultrasonography every 6-12 months may be useful for the early diagnosis of a mild form of nephrolithiasis.

### 10.2 VDDR1B

The best treatment for the patients with this form of rickets requires the association of calcifediol, as vitamin D has not/poor effect due to the altered hepatic conversion, and calcium supplements (**Table 6**). Serum 25(OH)D concentrations increased in response to a vitamin D bolus in heterozygous patients, although the achieved concentrations were lower than those found in controls. Homozygous patients with mutations in *CYP2R1* showed a very limited or no increase in serum 25(OH)D concentrations in response to the same vitamin D bolus (62, 96). Alternatively, other vitamin D active metabolites, such as calcitriol or alfacalcidol, may be effective in improving serum calcium and PTH concentrations.

### 10.3 VDDR2A and VDDR2B

The success of the treatment of patients with VDDR2A varies according to the VDR mutation (97). It has been found that mutations in the ligand-binding domain are associated with variable resistance to 1,25(OH)_2_D; whereas, mutations in the DNA binding domain cause almost total 1,25(OH)_2_D resistance and alopecia (2). Patients with alopecia are usually less responsive to treatment in comparison with patients without alopecia (98). However, the response to treatment is highly variable and the doses of vitamin D metabolites and calcium salts should be individualized. About half of patients with alopecia are resistant even to the highest vitamin D metabolites doses. The other half require ten times higher vitamin D metabolites doses than patients without alopecia (21, 78).

Levine (21) suggested that maintenance therapy is based upon several factors: (a) some patients will respond to calciferols (vitamin D_2_, vitamin D_3_, or calcifediol), which are substrates for the generation of 1,25(OH)_2_D; (b) other patients may respond only to high doses of vitamin D metabolites (calcitriol or alfacalcidol) that possess 1α-hydroxylation; (c) a minority of patients will not respond to calciferols Some patients affected by severe forms of the disease required long-term intravenous calcium administration (given over 12 h, often during the night) for many months in order to achieve normocalcemia (21, 78, 99).

During and after puberty, the patients may develop an increased intestinal calcium absorption to concentrations that are even greater than those of normal individuals. Indeed, some patients maintain normocalcemia with modest oral calcium supplementation, and a small number of patients may have normocalcemia even without calcium supplements (21).

Management of patients with VDDR2B is similar to that indicated in patients with VDDR2A (21, 99).

### 10.4 VDDR3

Few data on the treatment of patients with VDDR3 are available. These patients may respond to vitamin D or vitamin D metabolites, but greater doses are required in comparison within patients with nutritional vitamin D deficiency rickets. Lifelong treatment with a high dose of vitamin D are needed to ensure clinical and biochemical remission (21, 22). Some suggestions for the treatment of these patients are reported in **Table 6**.

## 11 Treatment and management of hypophosphatemic rickets

### 11.1 FGF23-dependent hypophosphatemic rickets

Conventional treatment of patients with FGF23-dependent hypophosphatemic rickets, including XLH, ADHR, ARHR1, and ARHR2, consists of inorganic oral phosphate salts combined with vitamin D active metabolites, such as calcitriol or alfacalcidol. Most of the data on the use of conventional treatment in hypophosphatemic rickets comes from the management of patients with XLH. The recommended starting and maintenance doses of inorganic phosphate salts and vitamin D active metabolites in patients with hypophosphatemic rickets (2, 100–103) are summarized in **Table 7**. Conventional treatment may be associated with a transient increase in serum phosphate concentration only in some patients, without significant changes in TmP/GFR. The treatment started in early infancy improves outcomes even if it did not completely normalize skeletal abnormalities (2, 104–106). It is generally associated with slow improvement in the healing of rickets and with residual skeletal deformities in most patients. Moreover, it has been shown that patients with XLH treated with conventional treatment had decreased height gain by 1 year of age and remained below population norms thereafter (107). This suggests that XLH affects skeletal growth and bone mineralization from the earliest years of life and that conventional treatment may not improve the evolution of the disease.

**Table 7:** Starting and maintenance doses of inorganic phosphate salts and active vitamin D analogs in patients with hypophosphatemic rickets.

	Newborns or before the development of clinical or radiological signs of rickets	Evidence of clinical or radiological signs of rickets
Starting doses	Alfacalcidol, 25-40 ng/kg/day (0.8-1 μg/day), once/day; inorganic phosphate salts, 20-40 mg/kg/day, 4 to 6 intakes/daily	Alfacalcidol, 40-80 ng/kg/day (1-1.5 μg/day), once/day calcitriol, 20-40 ng/kg/day, 2-3 times/day; inorganic phosphate salts, 40-60 mg/kg/day, 4 to 6 intakes/daily
Maintenance doses	Alfacalcidol, 25-40 ng/kg/day (1-2 µg/day), once/day; calcitriol, 20-30 ng/kg/day, 2-3 times/day; inorganic phosphate salts, 20-60 mg/kg/day, 4 to 6 intakes/daily	

N.B.: A progressive increase in the dose of phosphate supplements and active vitamin D analogs is recommended. The treatment should be personalized and adapted to the severity of the disease and patient’s tolerance. The two medications must be administered in combination and at balanced dosages and monitored carefully.

Adapted and modified from Carpenter TO, et al. (2), Baroncelli GI, et al. (53), Linglart A, et al. (100), Haffner D, et al. (101), Rothenbuhler A, et al. (102), Trombetti A, et al. (103), and authors’ expertise.

Conventional treatment may be effective in improving dental and parodontal lesions (100, 108, 109) depending on the onset, compliance, and duration of therapy, even though some studies demonstrated a poor effect of the conventional treatment on oral phenotype (110, 111).

In patients with ADHR and ARHR1 the dosages of phosphate supplements and vitamin D active metabolites may vary according to the severity of the disease and the response to treatment. In patients with ADHR, it has been shown that iron deficiency affected the severity of the phenotype, and that iron repletion normalized previously increased FGF23 concentrations and improved serum phosphate concentration (112). Therefore, reduction or withdrawal of conventional treatment should be attempted only after the optimization of iron status by oral iron administration (3 to 6 mg/kg elemental Fe, max 200 mg/day for 3 months) (113, 114). Measurement of ferritin is suggested to assess the need for iron supplementation in patients with ADHR (114).

Inorganic oral phosphate salts and vitamin D active metabolites constitute the conventional treatment also for patients with ARHR2 (115–117). However, the fact that ENPP1 deficiency may also be associated with arterial, cardiac, and articular calcification, or may present as generalized arterial calcification (115, 118, 119), should be taken into consideration because these phenotypes preclude the administration of the conventional treatment; therefore, the assessment of carotid intima media thickness and cardiac ultrasonography are strongly recommended in patients with ARHR2 (78). However, recent studies in patients with ARHR2 indicated that conventional treatment did not result in increased calcification in patients with ENPP1 deficiency (120, 121). Furthermore, patients with biallelic *ENPP1* mutations may develop normocalcemic primary hyperparathyroidism, which may require partial parathyroidectomy (116).

In patients with a form of FGF23-dependent hypophosphatemic rickets, the benefit of the conventional treatment should be balanced with the potential risks of overtreatment (53). Compliance with the treatment is poor in many patients, mainly in infants, due to gastrointestinal symptoms, including diarrhea, bloody stools, and abdominal pain; compliance may improve by reducing the dose of oral phosphate salts (53). Some complications have been reported in patients with XLH during conventional treatment, including hypercalcemia, secondary/tertiary hyperparathyroidism, hypercalciuria, nephrocalcinosis, and nephrolithiasis (53, 100–103). Some recommendations for follow-up in patients with XLH receiving conventional treatment have been suggested (2, 53, 100–103, 122) (**Table 8**).

**Table 8:** Some general recommendations for the follow-up of patients with XLH receiving conventional treatment.

Clinical assessment	Timing
Clinical and auxological examination^a^	< 5 years, 1-3 months; > 5 years: 3-6 months
Odontostomatology examination	Every 6-12 months^b^ or based on clinical symptoms
Orthopedic examination	Every 12 months^c^ or based on clinical symptoms
Otolaryngology	> 8 years or based on clinical symptoms
Biochemical parameters	
Serum calcium, phosphate, creatinine, alkaline phosphatase, PTH	Every 3-6 months
Urinary calcium, phosphate, creatinine^d^	Every 3-6 months
Imaging examinations	
Radiographs of wrists^e^, knees^e^, standing lower limbs^f^	Every 1-2 years or based on clinical signs
Renal ultrasonography	Every year^g^
Brain magnetic resonance imaging	In the presence of craniosynostosis or skull shape malformation, headache, neurological symptoms or visual disturbances
Quality of life	Every year^h^

^a^Including measurement of height, weight, body mass index, pubertal stage, intercondylar and intermalleolar distance, head circumference with skull shape; presence of signs of rickets, pain, stiffness, fatigue; neurological evaluation (in patients with craniosynostosis or spinal stenosis).

^b^After tooth eruption.

^c^After initiation of walking.

^d^To assess urinary calcium excretion as calcium/creatinine ratio or calcium excretion 24h, and TmP/GFR.

^e^To assess the rickets severity score when appropriate.

^f^To assess and quantify the degree of varism or valgism.

^g^Every 6 months if nephrocalcinosis is diagnosed.

^h^Assessed by using age-appropriate and disease-appropriate scales for children and adolescents.

Adapted and modified from Carpenter TO, et al (2),, Baroncelli GI, et al. (53), Linglart A, et al. (100), Haffner D, et al. (101), Rothenbuhler A, et al. (102), Trombetti A, et al. (103), Rainmann A, et al. (122), and authors’ expertise.

#### 11.1.1 Burosumab treatment in patients with XLH

Burosumab, a recombinant human IgG1 monoclonal antibody that targets FGF23, has been approved by the US Food and Drug Administration (FDA), the European Medicines Agency (EMA), and the Japan’s Health Authority (PMDA) for the treatment of patients with XLH. In Europe, burosumab is indicated in patients in children 1 year of age and older and adolescents with growing skeletons with radiographic evidence of rickets. The criteria for receiving burosumab vary between European countries. They are based according to eligibility rules for early access programs or qualification for reimbursement from health insurance or public health systems (123).

Some studies showed that burosumab treatment improved serum phosphate concentration and TmP/GFR values and were associated with rapid healing of radiographic sings of rickets, improved osteoarticular and muscular pain, and physical function (124, 125). Furthermore, a greater clinical improvement in rickets severity, longitudinal growth, and biochemical findings has been demonstrated in patients with XLH and an RSS of at least 2 treated with burosumab compared with patients continuing on conventional treatment (126). Beneficial effects of burosumab treatment on phosphate metabolism, skeletal lesions of rickets, and linear growth are reported in other recent studies (127–133). The main recommendations for treatment with burosumab for patients with XLH are summarized in **Table 9** (134–136). Serum phosphate concentration represents a main biochemical parameter to titrate the dose of burosumab. Dose adjustments should be continued until the target low-normal serum phosphate concentration is reached or no further increase is observed after dose escalation (101, 135). Likely, targeting fasting serum phosphate at the lower end of the normal reference range for age is the safest approach to reduce the risk of ectopic mineralization (101). Serial measurement of alkaline phosphatase activity may also have a pivotal role to assess the progression of disease (135). A progressive decline in serum alkaline phosphatase activity, despite still being above the age- and sex-specific upper limit of normal, may indicate a sufficient clinical response (135). Serum phosphate concentrations below the age- and sex-specific low limit of normal may be considered as a sufficient treatment response if is associated with a sustained decrease in serum alkaline phosphatase activity and improved signs of rickets (135). Periodic measurements of serum 25(OH)D concentration are suggested, because a decline in vitamin D status may be the consequence of reduced sunlight exposure but also to the effect of burosumab treatment (14, 134, 135). We recommend maintaining serum 25(OH)D >50 nmol/L (20 ng/mL) to prevent secondary hyperparathyroidism and to facilitate burosumab-mediated 1,25(OH)_2_D synthesis (135). Measurement of serum 1,25(OH)_2_D concentration during burosumab treatment is debated (14). A Consensus Statement recommended monitoring 1,25(OH)_2_D during burosumab treatment to assess the increment of this vitamin D metabolite (101), but its role in adjusting burosumab dose has not been documented. In the opinion of other authors, measurement of serum 1,25(OH)_2_D concentrations during burosumab adds cost without clear benefit, so it is not recommended (14).

**Table 9:** Main recommendations for the treatment with burosumab in patients with XLH.

• If guidelines and reimbursement criteria allow, burosumab treatment should be started as early as possible in patients over the age of 1 year (6 months in some countries, such as the USA), mainly in those with severe rickets (RSS ≥ 2). If conventional therapy is used as initial treatment, a prompt switch to burosumab is required in cases of insufficient response.
• Conventional treatment must be discontinued at least 7-10 days before starting burosumab treatment.
• A starting dose of burosumab of 0.8 mg/kg body weight, given subcutaneously every 2 weeks is recommended.
• Titrate burosumab in 0.4 mg/kg increments to increase fasting serum phosphate concentration within the lower end of the normal reference range for age to a maximum dosage of 2.0 mg/kg body weight (maximum dose 90 mg).
• Assess fasting serum phosphate concentration during the titration period between injections, ideally 7-11 days after the last injection, to detect the effect of treatment and to avoid hyperphosphataemia.
• After achieving a steady state of serum phosphate concentration, which can be assumed after 3-4 moonths on a stable dosage, fasting serum phosphate should be measured preferentially directly before injections to detect underdosing.
• Burosumab treatment should be discontinued if fasting serum phosphate concentrations are above the upper range of normal for age^a^.
• Serum phosphate concentration below the age- and sex-specific low-limit of normal may be acceptable if there is a sustained decrease in alkaline phosphatase activity, improvement of rickets, and the patient is responding clinically.
• Serum 25(OH)D concentration should be maintained at >50 nmol/L (20 ng/mL) to prevent secondary hyperparathyroidism and associated phosphaturia.
• Patients who have started treatment with burosumab should continue treatment throughout adolescence until the closure of the growth plate.
• Physiologic age-based parameters, such as growth plate closure, are likely the more appropriate indicators to determine when to change the burosumab dosing regimen from the pediatric (Q2W) to the adult dose (Q4W).
• A multidisciplinary evaluation should be conducted with the adult team during the transition phase to consider the follow­up of burosumab through adulthood.
• Changes in physical ability and quality of life in older patients may require the use of monitoring tools similar to those used in the adult population with XLH^b^.

^a^According to Summary of Product Characteristics (https://www.ema.europa.eu/en/documents/product-information/crysvita-epar-product-information_en.pdf) the next dose should be withheld and the fasting serum phosphate concentration reassessed in 2 weeks. The patient must have serum phosphate below the reference range before restarting burosumab. Once serum phosphate is below the reference range, treatment may be restarted at approximately half the previous dose, administered every 4 weeks. Serum phosphate concentration should be reassessed 2 weeks after any change in dose.

^b^e.g. 6-minute walk test, handgrip strength, Brief Pain and Fatigue Inventories and the Western Ontario and McMaster Universities Arthritis Index (WOMAC).

From Haffner D, et al. (101), Trombetti A, et al. (103), Seefried L, et al. (123), Padidela R, et al. (134), and Mughal MZ, et al. (135), and authors’ expertise.

Some recommendations for the follow-up of patients with XLH treated with burosumab are reported in **Table 10**. The timing of clinical evaluation and biochemical measurements may vary according to the expertise of the authors and should be tailored for each patient. Burosumab treatment is well tolerated and safe. The most common adverse drug reactions reported in pediatric patients were transient injection site reaction, headache, and pain in the extremities (101, 124, 132, 134–137).

**Table 10:** Some general recommendations for the follow-up of patients with XLH receiving burosumab treatment.

Clinical assessment	Timing
Clinical and auxological examination^a^	< 5 years: 1-3 months; > 5 years: 3-6 months
Odontostomatology examination^b^	Every 6-12 months or based on clinical symptoms
Orthopedic examination^c^	Every 12 months or based on clinical symptoms
Otolaryngology examination	> 8 years or based on clinical symptoms
Biochemical parameters	
Fasting serum calcium, phosphate, creatinine, alkaline phosphatase, PTH	Every 4 weeks during the first 3 months; thereafter at least every 3 months or based on clinical and biochemical pattern
Serum 25(OH)D and 1,25(OH)_2_D^d^	Every 3-4 months, mainly during the winter months
Urinary calcium, phosphate, creatinine^e^	Every 4 weeks during the first 3 months; thereafter at least every 3 months or based on clinical and biochemical pattern
Imaging examinations	
Radiographs of wrists^f^, knees^f^, standing lower limbs^g^	Every 1-2 years or based on clinical signs
Renal ultrasonography	Every 1-2 years^h^
Brain magnetic resonance imaging	In the presence of craniosynostosis or skull shape malformation, headache, neurological symptoms or visual disturbances
Quality of life monitoring	Every 3-6 months up to 1-2 years^i^
Other	
Monitoring adverse events^j^	Continuous active collection and reporting

^a^Including measurement of height, weight, body mass index, pubertal stage, intercondylar and intermalleolar distance, head circumference with skull shape; presence of signs of rickets, pain, stiffness, fatigue; neurologic evaluation (in patients with craniosynostosis or spinal stenosis).

^b^After tooth eruption.

^c^After initiation of walking.

^d^Adequate body stores of 25(OH)D may also facilitate burosumab-mediated 1,25(OH)_2_D synthesis.

Measurement of serum 1,25(OH)_2_D concentrations is not recommended by some authors (14).

^e^To assess urinary calcium excretion as calcium/creatinine ratio or calcium excretion 24h, and TmP/GFR.

^f^To assess the rickets severity score, when appropriate.

^g^To assess and quantify the degree of varism or valgism.

^h^Every year if nephrocalcinosis is diagnosed in patients who had received conventional treatment.

^i^Assessed by using age-appropriate and disease-appropriate scales for children and adolescents. The timing of evaluation may vary according to the clinical symptoms.

^j^In many European Countries healthcare professionals are asked to report any suspected adverse reactions to specific national databases.

Adapted and modified from Haffner D, et al. (101), Trombetti A, et al. (103), Rainmann A, et al. (122), Padidela R, et al. (134), Mughal MZ, et al. (135), and authors’ expertise.

#### 11.1.2 Growth hormone treatment in patients with XLH

Some studies showed that growth hormone (GH) in association with conventional treatment may increase short-term linear growth in patients with XLH (53). However, the effects on final height are not conclusive (138–140). A recent large study suggested that continuing GH treatment in patients with XLH after switching from conventional therapy to burosumab, if the height prognosis was compromised, might be beneficial for the final height (141). Nevertheless, GH treatment in association with conventional treatment (101) or burosumab is not recommended routinely, and further longer studies are required to examine the role of GH in addition to burosumab.

### 11.2 Other forms of FGF23-dependent hypophosphatemic bone disorders

Hypophosphatemic rickets may be a rare complication in some patients with non-lethal Raine syndrome (ARHR3) (78, 142–144) and in patients with fibrous dysplasia (145, 146). Hypophosphatemia has been reported in approximately 50% of patients with fibrous dysplasia/McCune Albright syndrome and is associated with rickets or osteomalacia. Serum FGF23 concentrations are increased due to a mass of FGF23-producing cells in fibrous bone lesions (145). Hypophosphatemic rickets may be treated with the conventional treatment by oral inorganic phosphate salts associated with vitamin D active metabolites.

TIO may be solved definitively by removing the tumor causing excessive production of FGF23 (23, 78, 147). Measurement of circulating FGF23 concentration may be useful to detect the progressive reduction after tumor resection or tumor recurrence (78). When tumor resection is not possible or the tumor is not found, and in patients awaiting surgery, conventional treatment similar to that used in patients with XLH should be administered (23, 24, 147).

#### 11.2.1 Burosumab treatment in patients with other FGF23-dependent hypophosphatemic bone disorders

Burosumab has been administered in patients with other forms of rickets, including two adult patients with ARHR1 who showed normalization of serum phosphate concentrations, healing of pseudofracture, reduced fatigue and bone pain (148), and in two children with fibrous dysplasia (149, 150) whose serum phosphate concentrations normalized (149) and alkaline phosphatase activity progressively reduced (150). Furthermore, some case reports in pediatric patients with cutaneous skeletal hypophosphatemia syndrome (27–30) showed that burosumab improved phosphate metabolism, bone turnover, skeletal abnormalities, and quality of life, suggesting that it could be a promising treatment for patients with the FGF23-mediated hypophosphatemia associated with this rare disorder.

### 11.3 FGF23-independent hypophosphatemic rickets

Monotherapy with oral inorganic phosphate supplements is the usual treatment for patients with HHRH, whereas active vitamin D metabolites are contraindicated due to the increased 1,25(OH)_2_D concentration (2, 151, 152). Treatment with phosphate should be targeted to decrease the 1,25(OH)_2_D concentration, reduce intestinal calcium absorption and hypercalciuria, and improve bone mineralization (2). Potassium citrate, salt restriction, and hyperhydration may be useful tools to reduce hypercalciuria. Moreover, treatment with fluconazole (100 mg once daily) has been proposed to inhibit the 1α-hydroxylase and reduce 1,25(OH)_2_D concentration and hypercalciuria to almost normal values (153). Furthermore, GH treatment associated with oral phosphate supplements improved the renal phosphate leakage and resulted in accelerated linear growth (154). The addition of GH, fluconazole, and salt restriction improved the effect of the conventional therapy with phosphate supplementation and potassium citrate (155). However, additional studies are required to provide the long-term efficacy and safety of fluconazole and GH alone or in combination.

Phosphate supplements and vitamin D active metabolites are suggested for the treatment of patients with hypophosphatemic rickets secondary to tubulopathies, Fanconi syndrome, Dent disease, or other systemic diseases, in association with the recommended treatments for each disease (2).

## 12 Conclusions

Rickets should be considered a health priority in infants and children. A correct clinical and biochemical approach is fundamental to identify the subjects with suspected rickets. Early diagnosis and adequate treatment are primary targets to avoid severe consequences later in life. The differentiation between the nutritional forms from the genetic forms of rickets is an important step for the correct treatment. Each form of rickets requires a specific diagnostic trail, distinct treatment and an interdisciplinary approach to management (53, 103, 123). Some algorithms to facilitate the diagnostic approach are suggested. Furthermore, some recommendations for the most appropriate treatments for patients with rare forms of hereditary rickets are proposed.

## Author contributions

GB: Conceptualization, Data curation, Formal analysis, Funding acquisition, Investigation, Methodology, Project administration, Resources, Software, Supervision, Validation, Visualization, Writing – original draft, Writing – review & editing. PC: Conceptualization, Data curation, Formal analysis, Investigation, Supervision, Validation, Visualization, Writing – original draft, Writing – review & editing. TA: Writing – original draft, Writing – review & editing. FB: Writing – original draft, Writing – review & editing. AC: Writing – original draft, Writing – review & editing. MC: Writing – original draft, Writing – review & editing. MC: Writing – original draft, Writing – review & editing. LD: Writing – original draft, Writing – review & editing. ND: Writing – original draft, Writing – review & editing. MF: Writing – original draft, Writing – review & editing. DF: Writing – original draft, Writing – review & editing. RF: Writing – original draft, Writing – review & editing. MK: Writing – original draft, Writing – review & editing. SL: Writing – original draft, Writing – review & editing. MM: Writing – original draft, Writing – review & editing. MP: Writing – original draft, Writing – review & editing. AS: Writing – original draft, Writing – review & editing. DT: Writing – original draft, Writing – review & editing. FV: Writing – original draft, Writing – review & editing. MW: Writing – original draft, Writing – review & editing. GW: Writing – original draft, Writing – review & editing. SM: Conceptualization, Data curation, Formal analysis, Funding acquisition, Investigation, Methodology, Project administration, Resources, Software, Supervision, Validation, Visualization, Writing – original draft, Writing – review & editing.
